# Individual placement and support in young people with severe mental illness: an Italian experience

**DOI:** 10.1192/j.eurpsy.2022.1934

**Published:** 2022-09-01

**Authors:** L. Pelizza

**Affiliations:** AUSL di Parma, Department Of Mental Health, Parma, Italy

**Keywords:** individual placement and support, psychiatric rehabilitation, mental health care, supported employment

## Abstract

**Introduction:**

Individual placement and support (IPS) has a considerable body of evidence for its effectiveness in helping people with mental disorder to obtain and maintain competitive jobs in the labour market. IPS closely follows 8 main principles (such as it aims to get people into competitive employment, it is open to all those who want to work, it tries to find jobs consistent with people’s preferences, it works quickly, it brings employment specialists into clinical teams, it provides time unlimited, individualised support, benefits counselling is included). However, little data in young adults are currently available, especially in Europe.

**Objectives:**

Aim of this study was to evaluate the beneficial effect of IPS in Italian young adults with severe mental illness, examining the main competitive employment outcomes and drop out rates during a 3-year follow-up period.

**Methods:**

54 participants were recruited from patients receiving psychiatric treatment in adult Community Mental Health Centers of an Italian Department of Mental Health. Together with drop out rates, we examined job acquisition, job duration (total number of days worked), total hours per week worked and job tenure (weeks worked on the longest-held competitive job).

**Results:**

A crude competitive employment rate of 40.7% and a crude drop out rate of 22.2% over the 3-year follow-up period were found. However, 66% of 42 clients who remained in the program over 3 years gained competitive employment at some time during the 3-year period.

**Conclusions:**

This research shows the feasibility of an IPS intervention model in the public mental health care system in Italy, especially for a young adult target population.

**Disclosure:**

No significant relationships.

